# Rapid Onset of Mesalazine‐Induced Eosinophilic Pneumonia Manifesting 7 Days After Initiation: A Case Report

**DOI:** 10.1002/rcr2.70758

**Published:** 2026-09-16

**Authors:** Toshiaki Inazaki, Kenichiro Takeda, Mana Iwasaki, Hiroki Tajima, Yu Shionoya, Ryutaro Hirama, Shun Sato, Akira Naito, Takeshi Kawasaki, Jun Ikari, Satoko Kageyama, Jun‐ichiro Ikeda, Takuji Suzuki

**Affiliations:** ^1^ Department of Respirology, Graduate School of Medicine Chiba University Chiba Japan; ^2^ Health Professional Development Center Chiba University Hospital Chiba Japan; ^3^ Department of Diagnostic Pathology, Graduate School of Medicine Chiba University Chiba Japan; ^4^ Department of Pathology Chiba University Hospital Chiba Japan

**Keywords:** drug‐induced lung injury, eosinophilic pneumonia, mesalazine, transbronchial lung cryobiopsy, ulcerative colitis

## Abstract

Mesalazine is a recognised cause of drug‐induced eosinophilic pneumonia. The condition usually occurs after 2–6 months of therapy, and onset within the first week is rare. A 29‐year‐old woman with bronchial asthma and newly diagnosed ulcerative colitis was initiated on oral mesalazine. On Day 7, she developed a persistent cough. Three weeks later, imaging revealed a right upper‐lobe pulmonary infiltrate, promoting referral for evaluation. Bronchoalveolar lavage and transbronchial cryobiopsy demonstrated marked eosinophilic infiltration, confirming eosinophilic pneumonia. Discontinuation of mesalazine led to rapid clinical and radiographic improvement. Clinical course and exclusion of alternative aetiologies identified mesalazine as the causative drug. This case demonstrates that mesalazine can induce eosinophilic pneumonia within 7 days of initiation.

## Graphical abstract

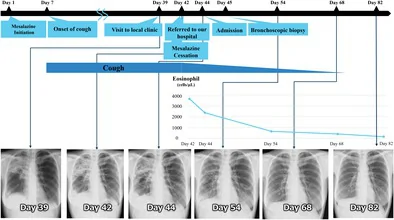

## 1 Introduction

Mesalazine, a 5‐aminosalicylic acid derivative, is widely used to treat inflammatory bowel disease, including ulcerative colitis and Crohn's disease [1]. Mesalazine is prescribed for both induction and maintenance therapy [2]. Drug‐induced eosinophilic pneumonia is a form of lung injury that occurs after exposure to a causative drug [3]. The condition is characterised by eosinophilic infiltration of the alveolar and interstitial spaces [3]. Bronchoalveolar lavage and transbronchial lung biopsy support the diagnosis of eosinophilic pneumonia [3].

Although mesalazine‐induced lung injury is uncommon [4, 5], mesalazine is frequently implicated in drug‐induced eosinophilic pneumonia [6]. Most cases occur after 2–6 months of treatment, and early‐onset cases are rare [7].

We report a case of rapid‐onset drug‐induced eosinophilic pneumonia where symptoms manifested 7 days after initiation of mesalazine and resolved without corticosteroid therapy. Clinical implications are discussed based on a review of the relevant literature.

## 2 Case Report

**FIGURE 1:**
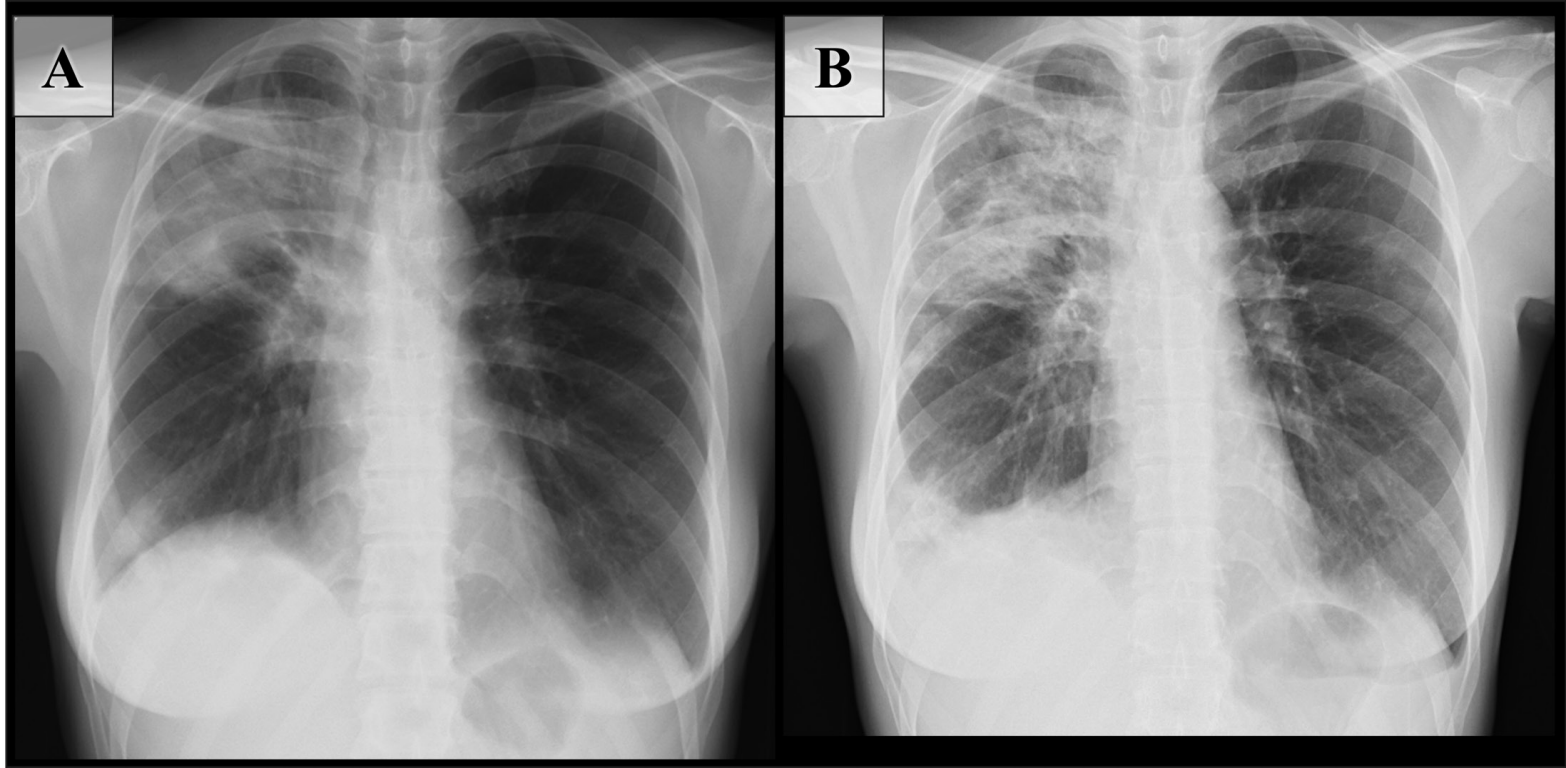
Comparison of chest radiographs. (A) Initial chest radiograph showing a consolidation in the right upper lung field. (B) Chest radiograph showing worsening pneumonia in the right upper lung and pleural effusion.

A 29‐year‐old Japanese woman was diagnosed with ulcerative colitis 1 month before presentation and was initiated on mesalazine (2.12 g/day). She had suspected bronchial asthma and used inhaled corticosteroids as needed. She reported no history of atopic diseases and had smoked a few cigarettes daily for 2 years. A productive cough developed 1 week after starting mesalazine. On day 39, follow‐up chest radiography showed consolidation in the right upper lung field (Figure 1A), and she was referred to our hospital.

**FIGURE 2:**
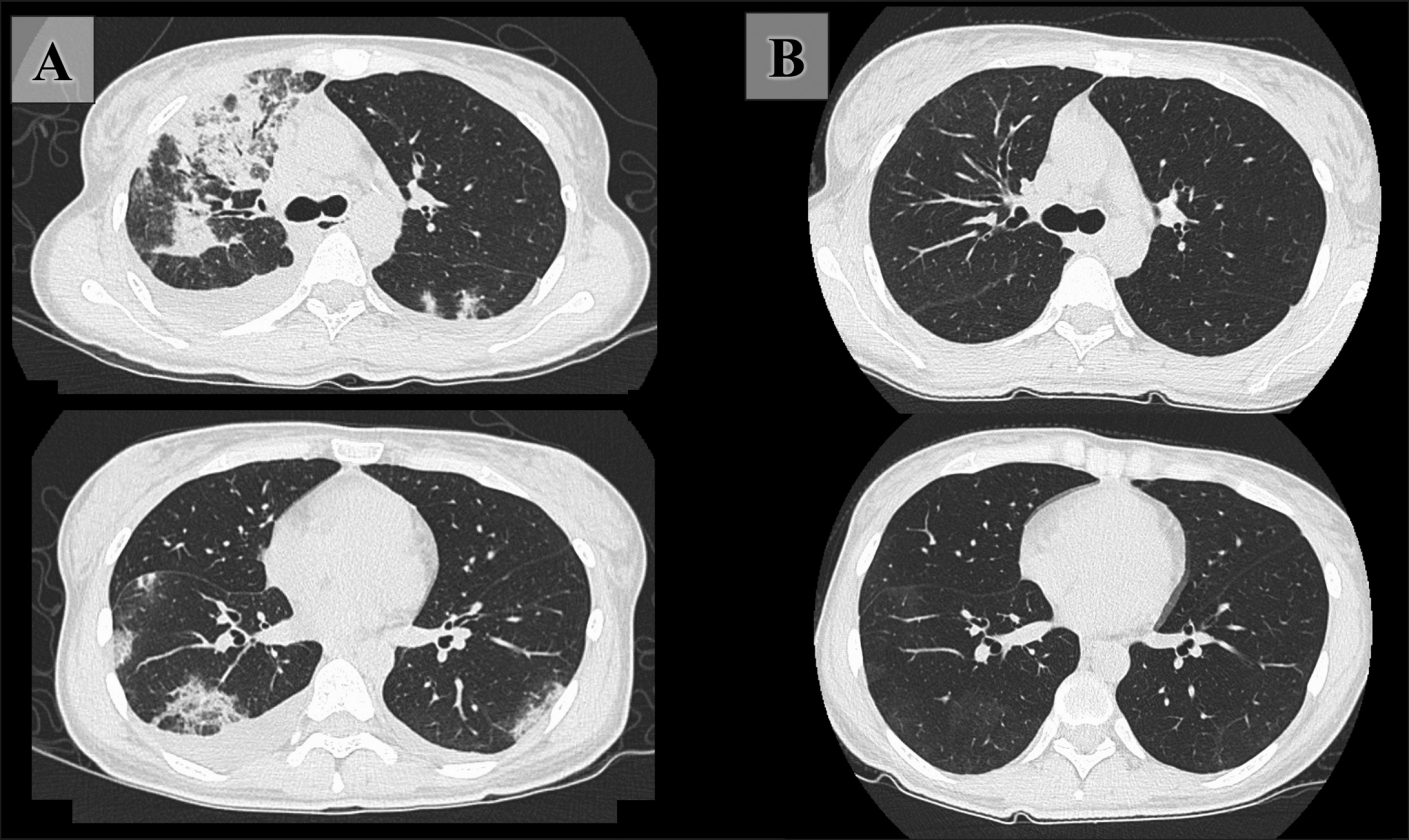
Chest CT images. (A) Chest CT image obtained at the first visit to our hospital showing diffuse consolidation, which was unilaterally predominant in the right upper lobe with pleural effusion. (B) A chest CT image obtained 42 days after stopping mesalazine showing no evidence of pneumonia.

Vital signs were stable: temperature 37.0°C, pulse 90 beats/min, respiratory rate 12 breaths/min, blood pressure 126/85 mmHg, and oxygen saturation 96% on room air. Breath sounds were clear bilaterally, and no skin rash or peripheral neuropathy was observed. Laboratory findings showed a white blood cell count of 9700/μL, with 37.8% eosinophils, and a C‐reactive protein level of 2.67 mg/dL. Serum immunoglobulin E levels were normal, and proteinase 3‐ANCA and myeloperoxidase‐ANCA were negative. Chest radiography showed progression of right upper‐lobe consolidation with pleural effusion (Figure 1B). Chest computed tomography demonstrated diffuse consolidation predominantly in the right upper lobe with pleural effusion (Figure 2A). Fractional exhaled nitric oxide was 51 ppb, indicating eosinophilic airway inflammation. Based on clinical history, peripheral eosinophilia, and imaging findings, mesalazine‐induced eosinophilic pneumonia was suspected.

**FIGURE 3:**
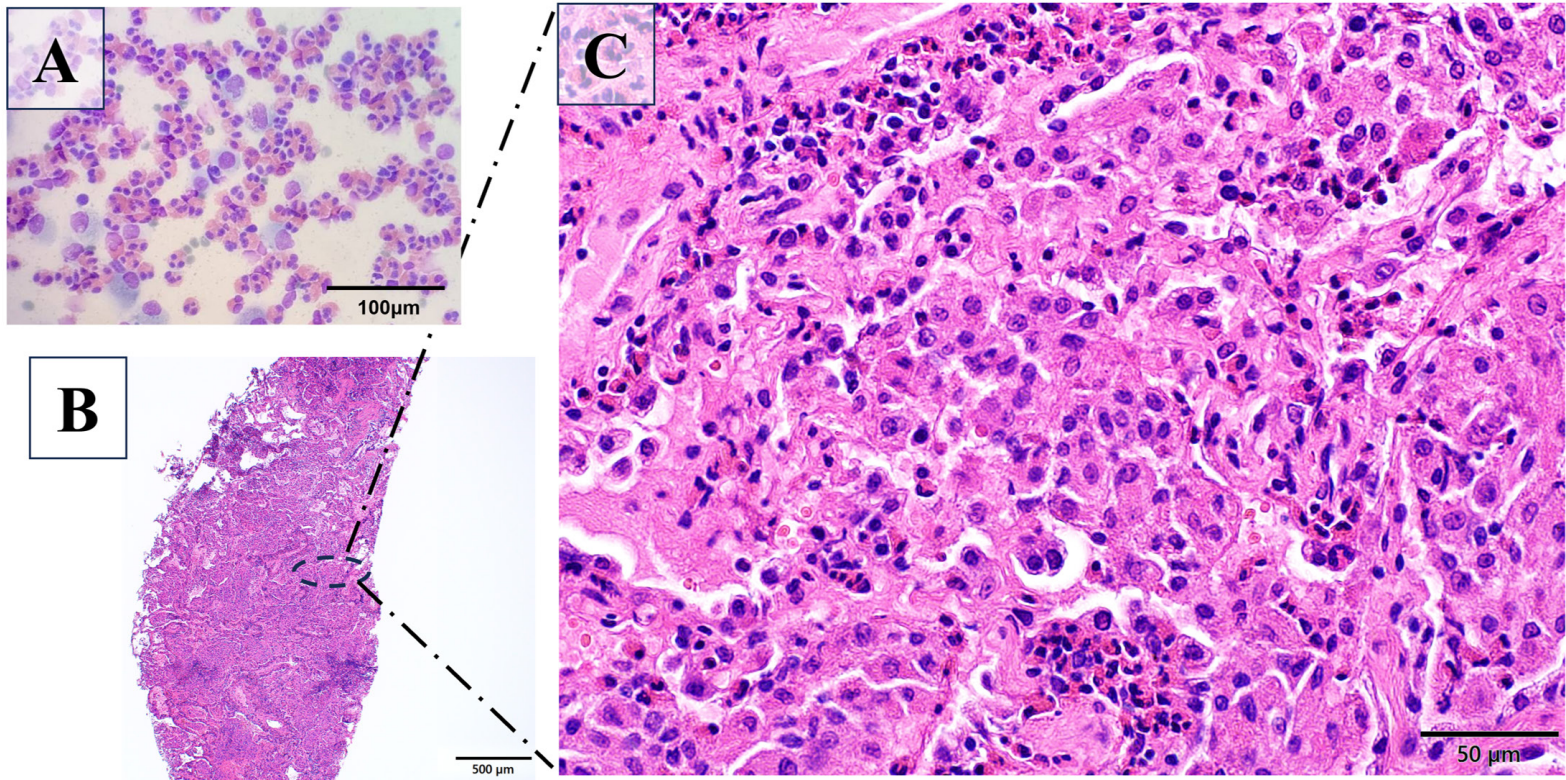
Pathological findings. (A) Cytological findings of bronchoalveolar lavage fluid showing numerous eosinophils (Diff‐Quick stain, ×40). (B) Histopathological findings of lung tissue obtained by cryobiopsy (haematoxylin–eosin (H&E) stain, ×4). (C) Histopathological findings of a cryobiopsy specimen showing eosinophilic infiltration in the lung tissue (H&E, ×40).

**FIGURE 4:**
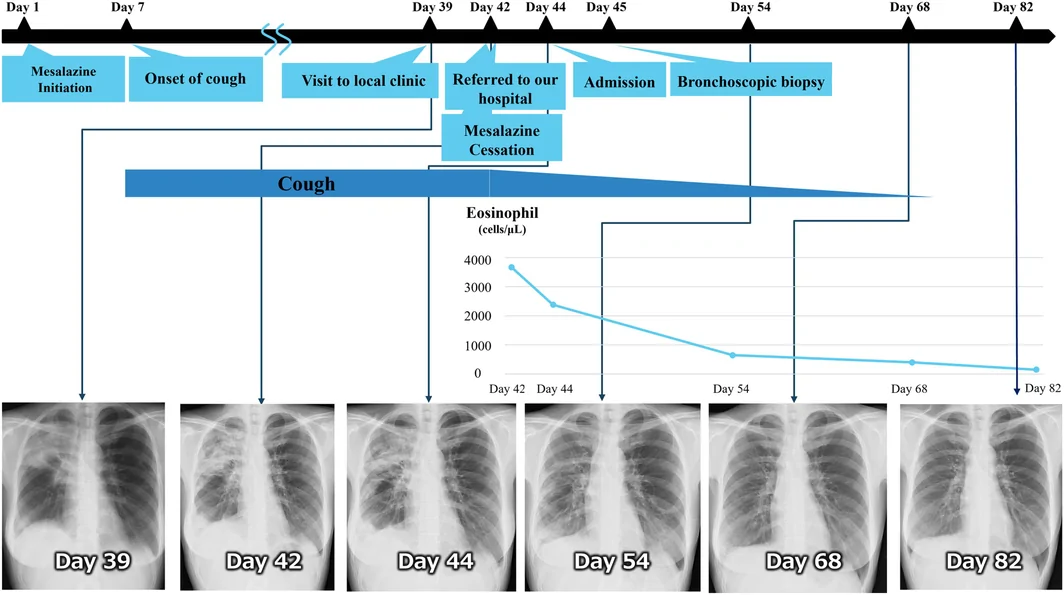
Clinical course. The horizontal axis shows the number of days after mesalazine initiation.

Mesalazine was discontinued, and the patient was admitted 2 days later for bronchoscopy. Bronchoalveolar lavage, transbronchial biopsy, and transbronchial lung cryobiopsy were performed. Bronchoalveolar lavage fluid recovery was 58 mL from 150 mL (38.6%). The total cell count was 5.88 × 10^6^/mL, with 78.6% eosinophils (Figure 3A). Transbronchial biopsy specimens showed dense eosinophilic infiltration in the alveolar spaces and interstitium (Figure 3B,C). Bacterial and mycobacterial cultures and cytology were negative. Based on the increased eosinophils in the bronchoalveolar lavage fluid and eosinophilic infiltration in the lung tissue, the patient was diagnosed with eosinophilic pneumonia. Chest radiography improved 2 days after mesalazine withdrawal (Figure 4), further supporting the diagnosis of drug‐induced eosinophilic pneumonia. Respiratory status remained stable, and no medication was administered. Her cough gradually resolved and disappeared within 1 month. Pulmonary infiltrates resolved 6 weeks after drug cessation (Figure 2B), accompanied by normalisation of peripheral eosinophil counts. This clinical course confirmed that eosinophilic pneumonia was induced by mesalazine. Vedolizumab therapy was subsequently initiated for ulcerative colitis without recurrence of eosinophilic pneumonia.

## 3 Discussion

This case highlights two clinical features: mesalazine can induce eosinophilic pneumonia soon after initiating treatment, and the reaction appears immune‐mediated rather than dose‐dependent.

In the present case, the patient developed symptoms 7 days after starting mesalazine. Previous reports describe symptom onset 1 month after treatment initiation [8]; therefore, this case represents an unusually early onset. A literature review was conducted using MEDLINE with the keywords “eosinophilic pneumonia” and “mesalazine”. Additional references cited in previous reviews were screened. Clinical variables, including age, sex, background disease, treatment duration, diagnosis, laterality, and treatment, were extracted. Fifty‐seven cases of mesalazine‐induced lung injury were identified in addition to the present case (Table 1) [1, 2, 4, 5, 7, 8, 9, 10, 11, 12, 13, 14, 15, 16, 17, 18, 19, 20, 21, 22, 23, 24, 25, 26, 27, 28, 29, 30, 31, 32, 33, 34, 35, 36, 37, 38, 39, 40, 41, 42, 43, 44, 45, 46, 47, 48, 49, 50, 51, 52]. Patient ages ranged from 14 to 84 years, with a peak incidence in the 30s. Twenty‐four patients were male, and 34 were female. Ulcerative colitis was the most common underlying disease. Among lung injury patterns, eosinophilic pneumonia accounted for 43%, consistent with previous reports [8]. Most patients exhibited bilateral pulmonary infiltrates, although unilateral disease occurred in some cases. Lung injury typically develops 2–6 weeks after treatment initiation. Onset within 2 weeks occurred in approximately 10% of cases, and onset within 10 days was reported in only two cases, including our patient. Corticosteroids are not always required for drug‐induced eosinophilic pneumonia. However, some reports describe prednisolone administration because of concerns about inflammatory bowel disease relapse after mesalazine discontinuation [5, 13]. Regarding the potential influence of bronchial asthma on lung injury, only one patient, in addition to our case, has been reported with bronchial asthma history [29]. In the previously reported case, the interval between mesalazine initiation and symptom onset was not described. In our patient, the elevated fractional exhaled nitric oxide level may have reflected pre‐existing eosinophilic airway inflammation associated with possible underlying bronchial asthma, rather than eosinophilic pneumonia alone. Although this finding raised the possibility that pre‐existing eosinophilic airway inflammation influenced the unusually early clinical presentation, any association between bronchial asthma and early symptom onset remains uncertain. An additional analysis of 18 well‐documented cases was performed to characterise mesalazine‐induced eosinophilic pneumonia (Table 2) [1, 2, 4, 5, 7, 12, 13, 14, 15, 16, 17, 18, 19, 20, 21, 22, 23]. Cases lacking detailed clinical data were excluded. Symptom onset ranged from 7 days to 14 months, with 70% occurring within 2 months. Our case represents one of the earliest reported symptom onsets, with symptoms appearing on day 7 after treatment initiation. The median cumulative mesalazine dose was 81 g, whereas the present case involved only 14 g. Previous studies suggest that eosinophilic pneumonia does not correlate with cumulative mesalazine dose [8], supporting an immune‐mediated mechanism.

**TABLE 1:** Literature review results.

Items	Results
Total cases, *n*	58
Age, median (range)	35 (14–84)
Male/female, *n* (%)	24 (41)/34 (59)
Background disease, *n* (%)	
Ulcerative colitis	49 (84)
Crohn's disease	8 (13)
Unknown	1 (2)
Duration of medication use, *n* (%)	
Within 2 weeks	6 (10)
2 ~ 6 weeks	18 (31)
6 ~ 12 weeks	10 (17)
Within 1 year	14 (24)
After 1 year	4 (7)
Unknown	6 (10)
Diagnose, *n* (%)	
EP	25 (43)
IP	16 (27)
OP	6 (10)
HP	3 (5)
Pleuritis	1 (1)
Unspecified	7 (11)
Laterality, *n* (%)	
Bilateral	39 (67)
Unilateral	5 (9)
Pleuritis	13 (22)
Unknown	1 (2)
Cases treated with corticosteroids, *n* (%)	
EP^a^	18 (69)
IP^b^	10 (63)
OP^c^	4 (66)
HP^d^	1 (33)

Abbreviations: EP, eosinophilic pneumonia; HP, hypersensitivity pneumonitis; IP, interstitial pneumonia; OP, organising pneumonia.

^a^ The number of cases with EP were 25.

^b^ The number of cases with IP were 16.

^c^ The number of cases with OP were 6.

^d^ The number of cases with HP were 3.

**TABLE 2:** Characteristics of the 19 cases of mesalazine‐induced eosinophilic pneumonia.

No	Report (year)	Age	Sex	Background	Daily use	Duration	Cumulative dose	Laterality	Treatment
1	Honeyboune et al. (1994)	30	Female	UC	1600 mg	7 months	336 g	Bilateral	Discontinuation
2	Tanigawa et al. (1999)	35	Female	UC	1500 mg	1.5 months	63 g	Unknown	Discontinuation
3	Saltzman et al. (2001)	53	Female	UC	Unknown	4 months	Unknown	Unilateral	Steroid
4	Perez et al. (2003)	50	Male	UC	Unknown	2 months	Unknown	Bilateral	Steroid
5	Hakoda et al. (2004)	30	Female	UC	2250 mg	1 month	67.5 g	Bilateral	Steroid
6	Machida et al. (2011)	26	Male	UC	2250 mg	1 month	67.5 g	Bilateral	Steroid
7	Kim et al. (2014)	30	Female	UC	1000 mg	19 days	19 g	Bilateral	Steroid
8	Ferrusquia et al. (2015)	32	Male	UC	1500 mg	14 months	630 g	Bilateral	Steroid
9	Kikuchi et al. (2015)	74	Male	UC	3000 mg	1 month	90 g	Bilateral	Steroid
10	Akiyama et al. (2015)	45	Male	UC	2400 mg	3 months	216 g	Bilateral	Discontinuation
11		22	Male	UC	2400 mg	1 month	72 g	Bilateral	Discontinuation
12	Patel et al. (2015)	16	Female	UC	Unknown	2 weeks	Unknown	Bilateral	Steroid
13	Franco et al. (2016)	39	Female	UC	3600 mg	6 weeks	216 g	Bilateral	Steroid
14	Zhang et al. (2016)	56	Female	UC	1500 mg	1 month	45 g	Bilateral	Discontinuation
15	Yeo et al. (2017)	24	Male	CD	3000 mg	9 months	777 g	Bilateral	Steroid
16	Gupta eta al. (2017)	22	Male	UC	Unknown	6 months	Unknown	Bilateral	Steroid
17	Park et al. (2019)	35	Female	UC	Unknown	1 month	Unknown	Bilateral	Steroid
18	Kobari et al. (2018)	53	Male	UC	3600 mg	11 days	112 g	Bilateral	Steroid
19	This case (2024)	29	Female	UC	2120 mg	7 days	14 g	Bilateral	Discontinuation

Abbreviations: CD, crohn's disease; UC, ulcerative colitis.

Drug‐induced lung injury arises through two principal mechanisms: direct cytotoxicity to alveolar epithelial or endothelial cells and immune‐mediated inflammation [6]. Mesalazine‐induced lung injury likely reflects immune activation [6]. Mesalazine may induce a Th2‐dominant response, with cytokines such as IL‐5 promoting eosinophil recruitment and activation [53]. From a pharmacokinetic perspective, approximately 20%–30% of orally administered mesalazine and 10% of rectal formulations are systemically absorbed [54, 55]. Cases of eosinophilic pneumonia have occurred after rectal administration [4], suggesting that small systemic exposures may trigger hypersensitivity. Sulfasalazine, a prodrug metabolised into mesalazine and sulfapyridine in the gastrointestinal tract, also causes eosinophilic pneumonia. Although toxicity was previously attributed to sulfapyridine moiety, accumulating reports of mesalazine‐induced disease indicate that 5‐aminosalicylic acid itself may trigger the immune response.

In conclusion, eosinophilic pneumonia can develop early in the treatment course. Drug‐induced eosinophilic pneumonia should therefore be considered when respiratory symptoms or pulmonary infiltrates develop during mesalazine therapy.

## Author Contributions

T.I. is the guarantor of this manuscript and contributed to the manuscript writing. K.T. helped with the acquisition of data and the writing of the manuscript. M.I., H.T., and Y.S. collected and analysed the clinical data of the patients and critically reviewed the manuscript. R.H., S.S., A.N., T.K., and Jun Ikari helped in the acquisition of data and critically reviewed the manuscript; S.K. and Jun‐ichiro Ikeda made pathological diagnoses and provided pathological images; T.S. supervised the study and critically reviewed the manuscript. All the authors have read and approved the final version of the manuscript.

## Funding

This research was funded by AMED (JP223fa627003, 25mk0121256h0503, and 25mk0121310h0201); the Health and Labor Sciences Research Grants on Diffuse Lung Diseases from the Japanese Ministry of Health, Labor and Welfare (grant numbers 23FC1030) and on Intractable Respiratory Diseases and Pulmonary Hypertension Research Group, Japanese Ministry of Health, Labor and Welfare (grant numbers 23FC1031).

## Consent

The authors declare that written informed consent was obtained for the publication of this manuscript and the accompanying images and attest that the consent form used to obtain the patient's consent complies with the journal's requirements, as outlined in the author guidelines.

## Conflicts of Interest

The authors declare no conflicts of interest.

## Data Availability

The data that support the findings of this study are available from the corresponding author upon reasonable request.
